# Is the proportion of the third inter-space that a neuroma occupies a more relevant measurement than the size alone?

**DOI:** 10.1186/1757-1146-3-S1-O17

**Published:** 2010-12-20

**Authors:** Helen Milnes, Julian Pavier, Simon Stinchcombe

**Affiliations:** 1 Sherwood Forest Hospitals, Notts, UK

## Introduction

Previous research has failed to show a correlation between the size of a neuroma and the level of pain experienced. The aim of this study was to determine if the proportion of the inter-space that a neuroma occupies has an impact on the level of pain and disability experienced.

## Methods

Forty consecutive subjects with third inter-space neuromas identified on ultrasound were included. The size of the neuroma and the inter-space were measured using ultrasound by the same examiner. Neuroma diameter was measured non-weightbearing plantarly and the inter-space was measured from the dorsal aspect when weightbearing. The proportion of the space occupied by the neuroma was calculated. Visual analogue pain scores and the Manchester-Oxford foot pain and disability questionnaire were collected.

## Results

Analysis of the data collected failed to show any correlations between the proportion of the space the neuroma occupies with either of the outcome measures used. The hypothesis; the greater proportion a neuroma occupies within the inter-space the greater the level of pain and disability experienced can be rejected.

## Discussion

The proportion of the inter-space that is occupied by a neuroma does not correlate to the level of pain and disability experienced.

